# Immortal data: a qualitative exploration of patients’ understandings of genomic data

**DOI:** 10.1038/s41431-023-01325-9

**Published:** 2023-03-31

**Authors:** Kate Lyle, Susie Weller, Rachel Horton, Anneke Lucassen

**Affiliations:** 1 Clinical Ethics, Law and Society (CELS), Wellcome Centre for Human Genetics, University of Oxford, Oxford, UK; 2 Clinical Ethics, Law and Society (CELS), Primary Care Population Sciences and Medical Education, University of Southampton Faculty of Medicine, Southampton, UK; 3 Centre for Personalised Medicine, University of Oxford, Oxford, UK

**Keywords:** Ethics, Patient education

## Abstract

As ambitions to ‘mainstream’ genetic and genomic medicine in the UK advance, patients are increasingly exposed to information about genomic data. Unlike the results of many other medical investigations which are linked to the time of sample collection, genomic testing provides *immortal* data that do not change across time, and may have relevance for relatives and generations far beyond the patient’s own lifespan. This immortality raises new ethical challenges for healthcare professionals, patients and families alike, such as ensuring consent for possible future interpretations; determining when genomic data are best sought (at birth, on illness etc) and reinterpreted; and balancing the confidentiality of patients and duties of care towards others. This paper reports on qualitative work exploring the perspectives of patients and relatives participating in genomic testing, and suggests that their engagements with this immortality are shaped by: the contrast between the simplicity of sample provision and information gathered; understandings of heritability; and notions of genomic data as a collective resource. We discuss the implications this holds for practice and argue that the immortality of genomic data must take a more prominent position in patient and healthcare professional interactions.

## Introduction

Patients are becoming more familiar with data related to their genetic code shaping their care, as ambitions to ‘mainstream’ genetic and genomic medicine in the UK advance. Unlike many other medical investigations, the relevance of which are linked to the time that the data were collected, data from [germline] genomic testing are enduring. Genomic data are largely stable across the life course, so although interpretation may alter over time, the data themselves will not change. As we share our genetic code with our biological relatives, insights gained from one individual’s data may have relevance for relatives and generations far beyond their own lifespan [1]. Moreover, when contributed to research databases or academic literature, the data have a continuing role in the interpretation of variants found in others. In these ways, genomic data are permanent, but more than this they live on beyond the individual: they are *immortal data*.

From this immortality arises new ethical challenges for healthcare professionals, patients and families alike. For example, the lifelong relevance and the breadth of inferences that can be drawn from genomic data mean that ensuring consent is sufficiently informed for the myriad of possible future interpretations is difficult to achieve at the point at which the sample is taken [2–5]. The evolving knowledge-base causes challenges for laboratory staff in determining when patient data should be reinterpreted, and the implications this may have for clinical care [6]. The heritability of genomic data means healthcare professionals will sometimes need to balance protecting the confidentiality of some patients with duties of care towards others [2, 7]. Similarly, healthcare professionals and patients are often unclear about their roles and responsibilities with regards to informing relatives about relevant genomic information, each assuming that the information will be delivered by the other party [8]. At other times, patients may not be aware of the relevance of their genomic data to relatives [9]. Furthermore, the retention of genomic data itself is contentious, provoking debates about who may access it, what it may be used for, and who should be informed about any findings [10–14].

Given this range of ethical challenges, it is important that we bring the immortality of genomic data to the fore. Taking a qualitative approach, this article explores the perspectives of patients participating in genomic testing and their relatives, and suggests that engagements with this immortality are shaped by: i) the simplicity of sample provision; ii) understandings of heritability; and iii) notions of genomic data as a collective resource. We consider how understandings of this immortality affect participants’ notions of ‘genetic responsibility’ to past, present, and future generations. We argue that these understandings must take a more prominent position in patient and healthcare professional interactions.

## Methods

This article integrates findings from two research projects that form part of the Ethical Preparedness in Genomic Medicine (EPPiGen) programme. One project is a qualitative longitudinal (QLR) study following the experiences of those affected by the process and outcomes of genomic testing, and the other is a qualitative project exploring how and why variations in the genetic code are given the status of being ‘results’. Whilst each study has a different focus, both are united by an emphasis on understanding the lived experiences of patients and families.

### Participants and recruitment

Our aim was to garner a diverse range of experiences including those who had been offered WGS, or in a minority of cases targeted sequencing, for a rare disease or cancer, or were linked to someone who had. Accordingly, a purposive approach to sampling was employed. Participants included index cases (*n* = 9), those tested to aid a relative’s diagnosis (*n* = 10), and partners (*n* = 3). The accounts of all participants involved in both studies at the time of analysis were included. Both studies are ongoing.

The majority were recruited via their involvement in a study that sought to explore experiences of the UK-based 100,000 Genomes Project (100kGP). As part of this work, participants completed a postal survey, focusing primarily on issues of consent and confidentiality and on which a willingness to take part in future research could be indicated (see ref. [3]). Some participants were recruited via the new NHS Genomic Medicine service or through NHS genetics clinics with access facilitated by healthcare professionals who distributed study information. Snowball sampling was used to recruit significant others, for example life partners.

### Data generation

Data were generated separately for each study, albeit as part of the overarching EPPiGen work programme. Both projects employed semi-structured interviews designed to capture accounts of journeys through genomic testing. Questions were constructed to explore participants’ experiences of the process and their subsequent care, understandings of genetic information, decisions deliberated, support received, and implications for their wider lives. Participants were sent information sheets and consent forms in advance, and written consent was sought at the interview. Interviews were conducted individually by KL, SW, and RH.

Interviews completed prior to the start of the pandemic were conducted in-person, primarily within participants’ homes. From March 2020, all interviews were conducted remotely either online via Microsoft Teams or by phone. The in-person and online interviews were of similar length (approximately one hour) and were comparable in terms of the depth and richness (see also ref. [15]). A range of creative tools such as the construction of family trees, timelines, and visual representations of patient journeys were used within many of the interviews. All interviews were recorded and transcribed verbatim.

### Data analysis

Although data sets were initially analysed independently, ‘immortality of data’ was identified as a common theme in research analysis meetings. Researchers from both projects adopted a collaborative and reflexive approach to thematic analysis [16] to explore the ways in which understandings of the immortality of genomic data were present in or absent from participants’ accounts. The process was aided by the qualitative analysis software package, NVivo (v12 Pro). KL, RH and SW conducted multiple readings of the data. Each brought different subjectivities, substantive interests, and disciplinary backgrounds to the analytic endeavour. The process was iterative and recursive with codes refined based on collective discussions.

To gain a more holistic sense of participant’s narratives, case analysis was undertaken drawing on Neale’s [17] case analysis toolkit. Summaries focusing on the structure/plot and recurring or contradictory themes within the accounts were constructed for each participant. Through this case analysis, more implicit understandings of the enduring nature of such data were captured. These included conversations about data living on through other family members and heritable risks to future generations, as well as notions of future benefit to research through the accumulation of data.

### Ethical considerations

The NHS East Midlands – Nottingham 2 and the NHS South Central Hampshire Research Ethics Committees granted approval for the studies. The work was guided by an ethic of care with emphasis placed on the situated nature of research ethics [18]. The data have been anonymised and pseudonyms are used for participants throughout the paper.

## Findings

The pooled material comprised the accounts of 22 participants, 12 of whom participated in multiple in-depth interviews as part of our QLR work. Conceptualisations of the immortality of genomic data were present across participants’ narratives in varying degrees and absent in some. In what follows, we show how focusing on the physical process of taking the sample often obscures the implications that the subsequent data may hold far beyond this point. We move on to explore how the immortality of the data is revealed through participants’ notions of heritability and the value of the data as a collective resource. Attention to the ways in which genomic data live within and beyond the individual, guides participants towards more collective notions of self, positioning themselves-in-relation to past, present, and future generations. As Hallowell [19] observes, such positioning brings a sense of ‘genetic responsibility’, and for our participants this was visible through their desires to provide genetic information to their own families as well as patient communities and wider populations.

### Simplicity of sample provision distracting from the immortality of data

For many participants, when asked about their experiences of having a genomic test the physical process of giving a sample was more tangible than the subsequent delivery of genomic data from that sample. Many people recounted their experiences of having blood taken, sometimes contrasting the ease of this against their experiences of other physically invasive investigations. For example, Claire’s journey to a diagnosis of colon cancer was protracted and involved multiple visits to primary care, followed by invasive tests (such as a colonoscopy) and treatment. In comparison, she described the genomic test as straight-forward:

‘You know, simple blood tests at the hospital’ (Claire).

Richard had genome sequencing to find a diagnosis for the neurological problems he was experiencing, and he too spoke of the simplicity of the test:

‘I was OK with it. It’s only a blood test. I didn’t have a problem with it … I wouldn’t hesitate to do it again. You know, just go. It’s not as though it’s an invasive test…’ (Richard).

Various participants reflected on what might have happened in the time since they provided a sample for testing. While evidently envisaging ongoing analytic work, many referred back to the physical sample they had provided. Shirley, in particular, focused on the role of the physical sample in the continuing analysis, contemplating whether the sample from her deceased sister had been frozen:

They did ring me up to say that they couldn’t find the BRCA gene [but they are still looking for other findings]… How long has [sister] been gone? Two years? … So they must freeze [sister’s sample] so they can keep it. (Shirley)

Using the physical sample as a surrogate for the data derived from it was common throughout the interviews, but at certain points the link between sample and data was lost, and participants appeared to think of genomic testing in terms of the sampling process alone. This was most evident when discussing the risks of having a genomic test – the physical process of sampling often seemed to determine participants’ views of its ‘invasiveness’, rather than the possibilities for the data that might be generated from their sample. This is illustrated by Harry, whose attention focused on the physical risk the procedure posed to the body over any other considerations:

‘Whether they gave me information or not, I’m not too worried about it, it’s not going to hurt me to have a needle put in me and have some blood taken out.’ (Harry).

Focussing on the act of providing the physical sample was used by some participants to protect themselves from disappointment or anxiety about potential outcomes from their genomic test:

‘I remember saying to my mum, “Can you, do you want to do this?” and she was quite apprehensive about that … She didn’t want to hear anything about any results. She was just coming in because I needed her blood, and that was cool. She said “I’ll do that, that’s not a problem, but I’m not interested in whether they’re going to tell me I’ve got a predisposition to this, that and the other”’(Harry).

Here, the focus on the physicality of the test provides a distraction from the immortality of the data generated, and the ensuing anxieties about the findings the test may reveal. Yet this strategy may also result in participants being blindsided by unexpected findings in the future.

### Understanding immortality through heritability

Participants frequently related to the immortality of genomic data through notions of heritability, which were often coupled with a sense of ‘genetic responsibility’ [19]. Indeed, for many participants, the value of finding a genetic explanation for a condition did not come from the relevance such data might have for them, but was linked to the implications it might have for their relatives. William provides an example, and here his wife Maggie explains that had it not been for the potential implications for their children, William may not have pursued the genetic aspects of his neurological condition:

“You [William] only ever wanted to have yourself investigated because I pushed for it because we’ve got children. I think your preference at the time early on was that actually, “No, if I’ve got what my dad had I’ll just run with it, see how it works, I’d rather not know much info”. (Maggie)

Through Maggie’s account we can see William’s, and indeed Maggie’s own, sense of genetic responsibility towards their children. Encouraged by his wife, William’s sense-making of his condition, that was based on notions of himself-in-relation to the past generation, expands to position himself-in-relation to present and future generations. With this repositioning, the focus is no longer on what genomic testing will do for William, but what it might mean for his children and future generations. Maggie clearly shares this sense of genetic responsibility, and indeed Maggie is the driving force behind the genetic investigations. Hallowell [19] demonstrates that ‘at-risk’ women feel an obligation to manage genetic risks in their kin, and in our data we can see that sense of responsibility is also felt by those who are not at genetic risk themselves. Genomic findings gain significance for participants and other family members from the subsequent actions they may enable, such as reproductive decision making. For some participants, prenatal testing offered the potential to intervene in the immortality of the condition. For example, Sophie participated in WGS to aid the diagnosis of a range of symptoms in her youngest daughter, and here Sophie reflects on the potential implications for her older daughter’s future parenthood:

‘So, I think the same for [older daughter], if she is a carrier of something, that is already true and us being able to give her more information and more support and more preparation and more signposting can only be helpful’ (Sophie).

Sophie’s example highlights how genomic data lives within one daughter and may live within her sibling(s). Sophie expresses a sense of genetic responsibility to provide her children with as much information as possible. Her narrative suggests that she views genetic information as something fixed and ‘out there’ to be discovered; something that is ‘already true’, rather than something generated through testing and interpretation.

For others, such as Ashley, prenatal testing could provide an opportunity to alleviate the anxiety caused by the uncertainty around patterns of inheritance. As she explains, not knowing if her new-born son had inherited the same condition that affects her older daughter, had a significant impact on her experiences during that time:

‘It would be good to get a cause – something for the sake of having closure, and obviously for prenatal testing … when [son] was born, for instance I think had I known that he didn’t have what [daughter] has, I would have enjoyed having him more. At the time … I was constantly waiting for him to not be like [daughter] and it took me a long time to relax and accept the fact that things were going to be OK’ (Ashley).

In other circumstances, the value of a genetic finding comes from its potential to make a condition visible in family members at an earlier time, potentially before the onset of physical symptoms, as Clive describes:

‘… the benefit of the genetic study is that it would make it easier to identify people that have got the gene but haven’t got symptoms’ (Clive).

Here, this information would offer the advantage of enabling such relatives to undertake risk-reducing treatments, or adopt behaviours to prevent or mitigate the onset of symptoms. The potential value of this information was clear for Clive, and he had taken on responsibility for trying to contact distant relatives in his wife’s family to alert them to the potential hereditary condition in their lineage. Similar to Maggie in the example above, it is interesting to note that Clive feels a sense of genetic responsibility to family members to whom he is connected by marriage rather than by genetics. This supports our findings reported elsewhere that genetic data causes us to reconceptualise who we see as the ‘patient’ [1].

Conversely, other participants focused predominantly on the physical manifestation of the condition rather than its potential genetic causes. For example, Betty’s understanding of the immortality of the data was closely tied to the physical manifestation of disease and at times it was difficult to establish when she was talking about a genetic predisposition to cancer, and when she was talking about cancer itself. She positioned herself only in relation to family members who had a diagnosis of cancer and was confident that her 100kGP results would not be relevant to her children and sister as they had not ‘got it’ (although some of them were younger than she had been at the age of her cancer diagnosis). Consequently, Betty had not spoken in any detail to her family members about her participation in 100kGP and having genomic testing:

“[sister] knows I’ve had [cancer], but I haven’t told her about this [genome test]… I didn’t think it was important.” (Betty)

In contrast to Betty, Diane has talked to other family members about the relevance her genomic data may have for them. However, Diane reported that it did not hold the same salience for her relatives:

‘We’ve talked to other [family] members—my daughter, again it would have impacted on her if we’d found something…but it’s not the same for her. And our other family members, I should think they’ve forgotten all about it… They weren’t really sort of that interested’ (Diane).

The examples from Betty and Diane demonstrate the implications of not fully appreciating the immortality of genomic data in different ways. In some cases, it may mean that information is not disclosed to potentially ‘at risk’ relatives, while in others the salience of that information may not be apparent to relatives when it is disclosed. For Betty, it meant that she did not discuss potential genetic-risks with relatives for whom that information might be relevant. Whereas although Diane did appreciate the risks and attempted engage relatives in these conversations, without their own appreciation of immortality Diane’s relative were not able to take the risks on board.

In these ways, participants conveyed understandings of the immortality of genomic data through notions of inheritance, and how they positioned themselves and their genetic data in relation to other family members. Below we discuss how these were also visible in participants’ positioning in relation to wider populations.

### Understanding immortality through notions of genomic data as a collective resource

The immortality of genomic data featured, both implicitly and explicitly, in participants’ narratives about the value of genomic data as a collective resource. It was common for participants to express altruistic and/or sanguine sentiments about the wider benefits of their participation in projects such as the 100kGP:

‘… in the bigger picture we’re contributing to worldwide research, aren’t we?’ (Maggie).

‘ … my objective was really to be part of what I believe was really important medical research.’ (Charles).

Positioning themselves in relation to other patients and wider populations came with a sense of genetic responsibility to contribute their data to research. For some, the collective benefits to be gained outweighed any concerns they had about data usage at an individual level:

‘I do care about my data and things like that, I don’t mind if it’s used forever, I don’t mind if you pick it up and drop it, I don’t mind any of that because it’s advancing the medical science’ (Mary).

Those who were parents of children under investigation, such as Carol and Monique, positioned themselves in relation to other families in similar circumstances. Although they hoped to gain answers for the medical problems that their own children had experienced, their participation in 100kGP was also motivated by a sense of genetic responsibility to the wider patient community:

‘… not only did it help [my son], but I just felt it could help other people and young people in the future.’ (Carol).

‘… if by looking at his blood or his saliva or his whatever, you know, helps them to make new discoveries and come up with new things, I think that is really important, for him [son] and for families in a similar situation in 10 years’ time …’ (Monique).

For participants such as Lynn, an understanding of the immortality of genomic data was posited in terms of leaving a legacy:

‘…at the time having been given this news [cancer diagnosis] that it looked really aggressive I was thinking I might not be around, so yes I was just hoping my sample would be there and I would leave some sort of legacy.’ (Lynn).

This was particularly salient as Lynn was grappling with her own mortality.

The importance of pooling and storing genomic data was recognised by some. For instance, Thomas described how his daughter’s probable diagnosis had been established not through the identification of a variant, but in conjunction with the analysis of genomic data from other children:

‘they’ve now identified it, not because there is a change in the marker, but because they found children who have a common thread of that marker being changed’ (Thomas).

Similarly, Jason understood that successful genomic testing relied on other people also contributing ‘immortal data’, and articulated the genetic responsibility of the community to do so:

‘the data, by its nature and by its necessity, needs to be shared. Like, it can’t **not** be shared. For the progress to be made—to help find the diagnosis that you originally went into the project for.’ (Jason).

A few participants spoke of the evolving nature of analysis and interpretation, and in so doing positioned genomic data as immortal:

‘because the development of testing is something that’s [in the] early stages, they can’t actually rule it out completely, so you’re still wondering whether or not you’ve still got it, they just haven’t found it for whatever reason.’ (Claire).

‘Somebody else could come along in another ten years’ time and say, “Gosh there’s all this data here, let me go through it” … and find something completely different’ (Diane).

We have shown how patients’ understandings of the immortality of genomic data are shaped by the act of sample provision, and notions of the data living in and beyond them through inheritance and collective resources. We now discuss how this impacts on patients’ experiences of genomic medicine.

### Immortal data in clinical contexts

Above, we have demonstrated the ways in which patients’ understandings of the immortality of genomic data may bring feelings of genetic responsibility to past, present, and future generations. In doing so, we appeal to the concept of genomic contextualism. In contrast to arguments of genetic exceptionalism that seek to establish genomic data as definitively ‘different’, Garrison et al. [20] demonstrate that ‘*genomic tests both share characteristics with other types of medical tests and represent a combination of features that make them distinct*’ [p.51]. They advocate the notion of genomic contextualism to recognize that ‘*the contexts where genomic information will be used will determine what qualities of genomic information are salient to the case at hand*.’

Here, we argue that in the context of patient experience, the immortality of genomic data becomes the relevant feature that distinguishes it from other medical investigations, and it is this immortality which should be made explicit to patients. Indeed, the ways in which patients understand the immortality of genomic data has important implications for their experiences of genomic medicine and the ethical issues it may raise. For example, in focusing on the physical sample as a relatively non-invasive and simple procedure, patients can lose sight of the myriad of possible diagnoses, tendencies, and predispositions that might be revealed (some of which may bring disadvantages in knowing about them). In these circumstances, patients may find themselves wholly unprepared for the types of information they receive through genomic testing. This may be particularly likely for people having tests primarily to facilitate the interpretation of another’s data, as their focus is directed towards the other and their own involvement may be obscured, as parents Jason and Steph illustrate:

Steph: I know we’re getting tested for all of the—whatever it is, I can’t remember, massive list. But I did more focus on the outcome for [daughter].

Jason: Yeah, it’s more like, we were a by-product

Steph: Yeah. Like we **had** to do that to help her and get her answers… I suppose we could get a bad result, couldn’t we? But you don’t really think about it.

Similarly, when patients view genomic tests as being like any other blood test that will reveal data with clear implications for their health and care, it can create the expectation that if nothing is found it means there is no underlying genetic cause for their condition. This can cause deep distress for patients on discovering the reality of their situation: that there may still be a genetic aspect to the condition, but that it has not [yet] been revealed. Maggie articulates well the distress she and her husband William experienced when they found themselves in this situation:

“We got our letter after two years saying, “Actually we haven’t found anything”—that felt a naïve relief at the time, because I was thinking, “Gosh, that means that they’ve confirmed that he hasn’t got what they said he’d got,” but when we [discovered it is genetic but that cause has not been found]…. That was a real… gut-sink—because there was a hope that maybe it’d be something else. But, it’s just an endless waiting game.” (Maggie)

While the argument that the sample often becomes conflated with the subsequent results could be made of any blood test, most other blood tests provide reasonably finite results, linked to particular medical conditions, that might be repeated over time as symptoms progress. In this setting, the conflation of samples, tests, and results is not so problematic. For genomic tests, the immortality of the data and uncertainty around its implications destabilises this relationship, and consequently this conflation can cause challenges for patients and their families.

## Conclusion

To navigate the challenges raised by genomic data effectively, it is essential to ensure that their immortality is brought to the fore in communications between healthcare professionals, patients, and relatives. At a practical level, we suggest that one positive step would be for healthcare professionals to consistently refer to ‘data’ over ‘samples’ in their communications with patients and relatives, and make explicit the relationship between the physical sample that will be taken and the subsequent data that will be generated. In doing so, healthcare professionals must pay attention to the subtleties of the difference in the relationship between sample, tests, and results for genomic tests as compared with other blood tests, to draw attention to the immortality of this data. Our findings indicate that patients and relatives conceptualise the immortality of genomic data through their understandings of the implications of the data for both family members, and wider society through collective resources. Therefore, focussing conversations around these issues may also be useful in highlighting the immortality of the data.

As the role of genomic data in healthcare expands, in the UK for instance through the NHS Genomic Medicine Service and the Newborn Genomes Programme, explicit discussions regarding the immortality of genomic data and its implications for patient care—both in the present, and how that may change with future re-interpretations—will become increasingly important. Indeed, exposing the immortality of genomic data is an essential aspect of facilitating consent for genomic testing. Further research is required to explore the salience of the findings of this research beyond the UK context.

## Data Availability

The data sets generated and analysed during the current study are not publicly available because of the ongoing nature of our studies, but will be offered to UK Data Archive once the studies are complete, and maybe available from the corresponding author on reasonable request.
